# All-sapphire-based high-temperature pressure sensor system with in situ temperature compensation: innovative cavity design, fabrication, and APSC-FFT algorithm

**DOI:** 10.1038/s41378-026-01290-5

**Published:** 2026-04-29

**Authors:** Jiahang Tan, Feng Qin, Ning Wang, Zhiqiang Shao, Jie Zhang, Yong Zhu

**Affiliations:** 1 College of Optoelectronic Engineering, Chongqing University, Chongqing, 400044 China; 2 The 49th Research Institute of China Electronics Technology Group Corporation, Harbin, 150000 China

**Keywords:** Electrical and electronic engineering, Optical sensors

## Abstract

Targeting the environment of gas-cooled reactors (800 °C, 1 Mpa), we propose an all-sapphire composite-cavity sensor system for pressure measurement with in-situ temperature compensation. The sensor based on a fully sapphire-based dual-cavity structure and white-light interferometry principle. Decoupling two cavities’ information enables the simultaneous measurement. The high hardness and excellent thermal stability of sapphire ensure the high-temperature resilience. The novel central platform structure of the pressure-sensitive diaphragm enhances the spectrum contrast and measurement accuracy while maintaining high sensitivity. An optimized MEMS wet etching process guarantees superior diaphragm surface roughness and etching rate, and high-temperature wafer-level bonding ensures hermeticity. Furthermore, the adaptive peak-shift correction FFT algorithm is proposed for demodulation, achieving a sub-nanometer theoretical resolution. Experimental results under 0–1.2 MPa and 28–800 °C demonstrate that the system exhibits systematic error better than 0.13% F.S (temperature) and 0.18% F.S (pressure). The stability is better than 0.04% F.S. (temperature) and 0.12% F.S (pressure). The sensing chip remains stable performance after prolonged annealing at 1500 °C followed by cooling. It demonstrates the sensor is suitable for pressure monitoring in 800 °C and the potential of the chip for applications in extreme temperature, such as exceeding 1300 °C in aero-engines.

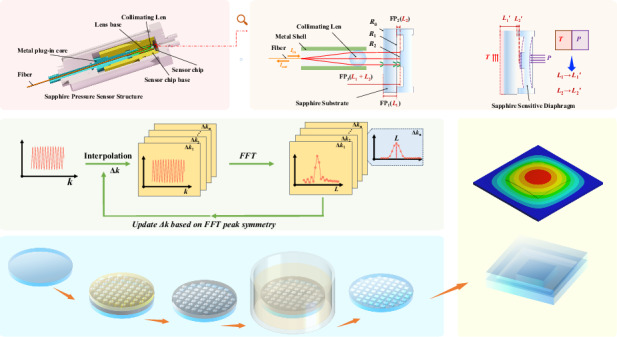

## Introduction

High-temperature pressure measurement represents one of the critical technical domains in extreme environment sensing^1^, exemplified by environmental monitoring in nuclear reactors, where the coolant (helium-xenon mixture) in gas-cooled reactors reaches 800 °C^2^, and condition monitoring of aerospace engines^3^, where the combustion chamber temperature in turbine engines exceeds 1300 °C^4^. Consequently, pressure sensors capable of operating in such extreme high-temperature environments are crucial for the advancement of fields like nuclear power and aerospace^5^. For the environmental conditions of gas-cooled reactors, specifically 800 °C and 1 MPa, traditional temperature measurement primarily relies on thermocouples and infrared techniques, which are significantly affected by environmental conditions and exhibit limited accuracy over the entire temperature range^6^. For high-temperature pressure sensing, electrical sensors such as those based on Silicon-On-Insulator (SOI)^7^, co-fired ceramic-based LC resonators^8^, and piezoelectric types are constrained by issues like piezoresistive coefficient degradation and electromagnetic interference under high temperatures^9^, leading to limitations in measurement range, temperature tolerance, or accuracy. Conversely, optical sensors like Fiber Bragg Gratings (FBGs) and fiber-optic Fabry-Perot (FP) sensors are widely employed in various extreme environment measurements due to their inherent advantages^10,11^, including immunity to electromagnetic interference, high resolution, and corrosion resistance^12^. In the field of pressure measurement, the configuration of a Fabry-Perot (FP) cavity formed between a pressure-sensitive diaphragm and a substrate surface represents a widely adopted approach due to its structural simplicity^13^. Employing diverse materials enables this configuration to meet the requirements of various applications, such as aerospace and healthcare. However, in high-temperature environments, severe pressure-temperature cross-sensitivity necessitates critical in situ temperature compensation^14^. Consequently, composite-cavity FP sensors capable of multi-parameter measurement have been proposed for decoupling parameters like temperature-pressure^15–18^, temperature-refractive index^19^, and temperature-humidity^20^. A rationally designed composite-cavity structure can achieve in situ temperature compensation for pressure measurements using FP sensors under high-temperature conditions. The typical composite-cavity FP pressure sensor comprises a substrate cavity and an air-gap cavity. The substrate cavity, being pressure-insensitive, serves for temperature measurement, while the air-gap cavity is dedicated to pressure measurement. The demodulation technique for the cavity gaps significantly impacts sensor performance. Current demodulation schemes for fiber-optic FP sensors primarily include peak-to-peak detection^21^, Fourier transform^22,23^, hardware correlation^24^, and spectral matching^25^, which exhibit an inherent trade-off between precision and processing speed. Currently, planar diaphragms represent the predominant sensing diaphragm type, differing primarily in shape, with sensitivity adjusted through thickness and dimensions. Achieving high-precision measurement demands high sensitivity from the sensing diaphragm. Nevertheless, excessive deformation of highly sensitive diaphragms degrades the quality of the FP reflection spectrum, subsequently reducing the accuracy of parameter extraction. Therefore, the design of the diaphragm structure is crucial.

The demanding performance requirements for sensor materials in extreme high-temperature environments make single-crystal sapphire an ideal candidate^26^, owing to its high hardness, excellent thermal stability, resistance to chemical corrosion, and broad optical transmission spectrum, coupled with a melting point as high as 2045°C. Currently, sapphire-based optical fiber sensors have been successfully applied to measure parameters like temperature and pressure in extreme environments, demonstrating outstanding performance^23,27^. The fabrication of sapphire-based optical Fabry-Perot sensors predominantly employs dry etching techniques. However, these methods are often characterized by low etch rates and excessively high surface roughness of the etched features, which severely degrades the overall performance of the sensor system^28^. Furthermore, advancements in MEMS wet etching and bonding techniques provide robust technological support for fabricating sensing structures from sapphire^22,29^. The sensing chip mandates a very high surface finish, making the etching process considerably challenging. Consequently, intensive research into etching methodologies and the optimization of critical process parameters is required. This research is essential to achieve precise control over etch time and depth, thereby guaranteeing superior etching quality.

We present an all-sapphire composite cavity optical fiber Fabry-Pérot (SCCOF -FP) high-temperature pressure sensor featuring a pressure-sensitive diaphragm with a pedestal-shaped structure. This pedestal design effectively ensures high sensitivity of the sapphire diaphragm, while the minimal deformation occurring on the pedestal platform further enhances the contrast of the interference fringes, thereby increasing the accuracy of the pressure measurement. The composite-cavity structure enables the simultaneous in-situ detection of temperature and pressure, as well as temperature compensation for the pressure measurement. To enhance both the accuracy and speed of sensor parameter extraction, this work proposes an adaptive peak-shift correction fast Fourier transform algorithm (APSC-FFT). This algorithm utilizes an adaptive resampling interval to rectify the peak shape in the Fourier transform, thereby reducing the peak position estimation error. The sensor diaphragm structure was fabricated using an optimized MEMS wet etching process, and the sapphire sensing chip was obtained via direct bonding technology. The sensing chip maintained excellent operational performance even after prolonged annealing at 1500 °C and subsequent cooling and packaging. Tests conducted on the fully packaged sensor over a temperature range of 28–800 °C and a pressure range of 0–1.2 MPa demonstrated a full-temperature-range pressure measurement accuracy of 0.18% F.S.

## Results

### SCCOF -FP sensing chip model

**Fig. 1:**
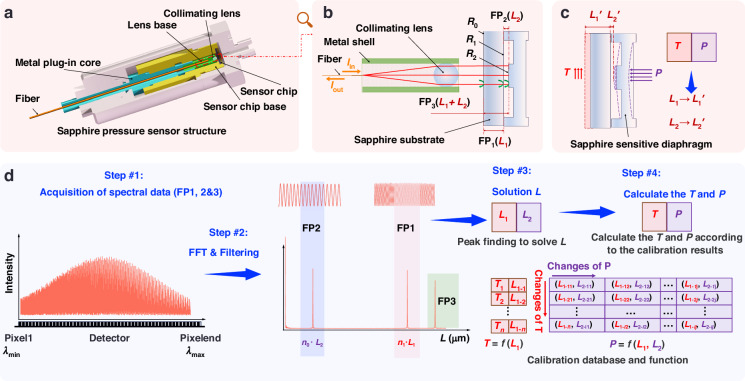
Diagram of the measurement mechanism of SCCOF-FP and its measurement process. **a** Structure of SCCOF-FP, **b** optical Model of SCCOF-FP, **c** diagram of SCCOF-FP sensing chip changing with T and P, **d** the demodulation process of SCCOF-FP

Figure 1a illustrates the fundamental structure of the proposed sensor, which comprises a gold-coated lead-in fiber, a collimating lens, a sapphire sensing chip, and a packaging structure. The sensing chip consists of a pressure-sensitive diaphragm and a substrate. The diaphragm is a pedestal-shaped sapphire structure fabricated using MEMS wet etching technology, which is then bonded to the sapphire substrate via direct bonding technology. As shown in Fig. 1b, the initial thickness of the sapphire substrate is designed to be *L*_1_ = 200 μm. The pressure-sensitive area within the pedestal-shaped diaphragm measures 3 mm × 3 mm, and the platform dimensions are 1.5 mm × 1.5 mm. The initial thickness of the air gap is designed to be *L*_2_ = 80 μm. Light from a broadband source is emitted from the fiber, collimated by the lens, transmitted through the substrate cavity, and incident upon the platform surface of the pedestal-shaped diaphragm, with a spot diameter of approximately 500 μm. The light beams reflected from the front and rear surfaces of the substrate cavity and the platform surface are collected by a spectrometer. The collected spectral data is processed by the algorithm to extract the cavity length information of the composite cavities. Subsequently, the temperature and pressure information is determined based on a calibration model. As shown in Fig. 1b, c, the sensing model incorporates three Fabry-Perot (FP) cavities. FP1 is formed by a monolithic sapphire substrate. Its uniform thickness and minimal, symmetric deformation under pressure render it virtually insensitive to pressure. Changes in temperature, however, alter its cavity length through the thermal expansion of sapphire. Conversely, FP2 is formed by the air gap between the bottom surface of the sapphire substrate and the pressure-sensitive diaphragm, making its cavity length susceptible to both pressure and temperature variations. By performing a sensor-specific calibration that establishes the relationship between the cavity lengths, temperature, and pressure, simultaneous measurement of these two parameters is achieved. (Details of the temperature compensation process are provided in Supplementary Information S4.)

The demodulation process is illustrated in Fig. 1d. The sensor’s reflection spectrum results from the superposition of light amplitudes reflected from each reflective surface of the sensing chip. For the all-sapphire chip with three reflective surfaces, the reflectivities are *R*_0_ = *R*_1_ = *R*_2_. Under the condition of low reflectivity, where only two-beam interference is considered, the reflected light intensity is given by:

1 $$\begin{array}{l}{I}_{\mathrm{out}}(\lambda )={R}_{0}{I}_{\mathrm{in}}+{(1-{R}_{0})}^{2}{R}_{0}{I}_{\mathrm{in}}+{(1-{R}_{0})}^{4}{R}_{0}{I}_{\mathrm{in}}+\\ {R}_{0}(1-{R}_{0}){I}_{\mathrm{in}}(2{\mathrm{cos}}(\frac{4\pi {n}_{1}{L}_{1}}{\lambda }-\pi ))+\\ {(1-{R}_{0})}^{3}{R}_{0}{I}_{\mathrm{in}}(2{\mathrm{cos}}(\frac{4\pi {n}_{0}{L}_{2}}{\lambda }+\pi ))+\\ {(1-{R}_{0})}^{2}{R}_{0}{I}_{\mathrm{in}}(2{\mathrm{cos}}(\frac{4\pi {n}_{1}{L}_{1}}{\lambda }+\frac{4\pi {n}_{0}{L}_{2}}{\lambda }))\end{array}$$

Where *I*_out_ is the reflected light intensity, *I*_in_ is the incident light intensity, *λ* is the wavelength, *L*_1_ is the thickness of the sapphire substrate, *n*_1_ is the refractive index of sapphire, *L*_2_ is the air-gap length, and *n*_0_ is the refractive index of air. The alternating-current(AC) component in the reflection spectrum model is a standard cosine function of the wavenumber *k* = 2π*n*/*λ*, whose frequency and phase contain the target gap value *L*. Transforming the intensity model into the wavenumber domain and simplifying yields:

2 $$\begin{array}{c}{I}_{{\rm{out}}}(k)={I}_{{\rm{dc}}}-{I}_{{\rm{ac}}\_1}\,\cos (2{n}_{1}{L}_{1}k)-{I}_{{\rm{ac}}\_2}\,\cos (2{n}_{0}{L}_{2}k)+\\ {I}_{{\rm{ac}}\_3}\,\cos (2{n}_{1}{L}_{1}k+2{n}_{0}{L}_{2}k)\end{array}$$

Where *I*_dc_ is the direct-current (DC) component from Eq. (1), and *I*_ac_1,2,3_ are the amplitudes of the AC components from Eq. (1). Each AC sub-term corresponds to the individual cavity reflection spectrum of FP_1_, FP_2_, and FP_3_, respectively. For simplicity, taking the cosine term corresponding to FP_1_ as an example, its Fourier transform result is:

3 $$\begin{array}{c}{F}_{ac\_1}(\xi )={\int }_{-\infty }^{+\infty }\cos (2{n}_{1}{L}_{1}k){e}^{-i2\pi \xi k}dk\\ =\frac{1}{2}{\int }_{-\infty }^{+\infty }({e}^{i(2{n}_{1}{L}_{1}k)}+{e}^{-i(2{n}_{1}{L}_{1}k)}){e}^{-i2\pi \xi k}dk\\ =\pi \delta (2{n}_{1}{L}_{1}-2\pi \xi )+\pi \delta (2{n}_{1}{L}_{1}+2\pi \xi )\end{array}$$

Where *F*_ac_1_ is the Fourier transform result of the cosine term for the FP_1_ reflection intensity, and *ξ* is the Fourier transform variable, which is related to *L*_1_. *F*_ac_1_ reaches its maximum at *ξ* = *n*_1_*L*_1_/*π*. Thus, *L*₁ can be directly obtained from the peak position using *L*_1_ = *ξπ/n*_1_. The result for FP_2_ is analogous. The correspondence between *L*_1_ -*L*_2_ and temperature (*T*)-pressure (*P*) is established through experimental calibration. Once *L*_1_ and *L*_2_ are calculated, the measured *T* and *P* values can be determined based on this calibration result.

### SCCOF -FP sensing chip structure

Most diaphragm-based pressure sensors employ planar diaphragms. When subjected to pressure, the pressure-sensitive diaphragm undergoes significant bending deflection, resulting in a decrease in the air-gap cavity length, while the substrate thickness remains constant. Under identical pressure, more pronounced deflection of the diaphragm corresponds to higher pressure sensitivity. However, in the sensing system illustrated in Fig. 1b, the reflected spectral intensity can be equivalently described as the integral of the reflected light intensity over the effective spot area of the collimated beam on the diaphragm surface.

**Fig. 2:**
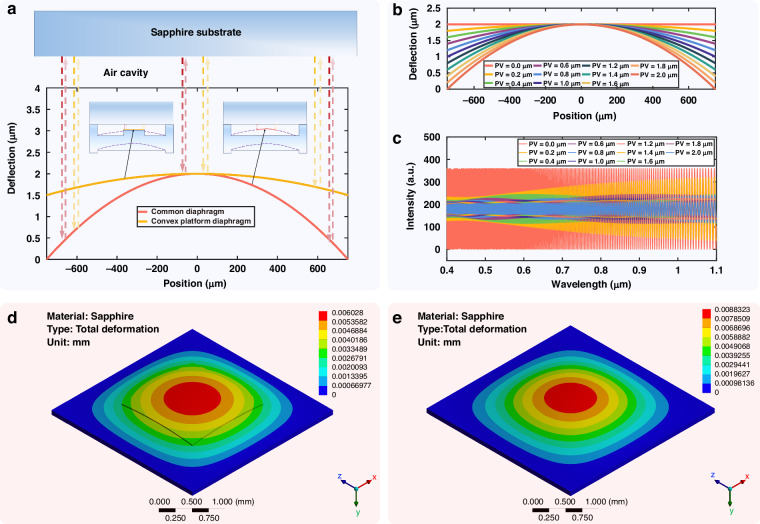
Diagrams of deformation in pressure-sensitive diaphragms. **a** Air cavity variation, **b** deformation at PV = 0–2 μm, **c** single-cavity reflection spectra under deformation at PV = 0–2 μm, **d** diaphragm with pedestal-shaped structure, **e** diaphragm of planar

As shown in Fig. 2a, severe deflection leads to substantial variation in the *L* across the integration area, which in turn causes a degradation in the spectral contrast. The extent of diaphragm deformation is quantified by the Peak-to-Valley (PV, *PV* = *L*_max_ - *L*_min_) value of *L* within the effective spot area, as illustrated in Fig. 2b, c. Reflection spectra of a single cavity were simulated for an initial air gap of 80 μm, with the *PV* variation modeled using a quadratic curve. The simulations demonstrate that the quality of the reflection spectrum deteriorates rapidly as the *PV* increases (Details of the reflection spectra are provided in Supporting Information S1). Furthermore, the extracted cavity length *L* corresponds to the average gap length within the area. A larger *PV* value inevitably leads to a greater absolute error in the calculated value of *L*.

To further enhance the sensor’s accuracy, a pedestal structure was incorporated onto the planar diaphragm (as shown in Fig. 2e) at the central light-sensitive region. This design aims to reduce the deformation within the effective spot area (i.e., a decreased *PV*) while maintaining the overall sensitivity of the diaphragm. The Finite Element Method (FEM) simulation results, presented in Fig. 2d, e, demonstrate that under a uniformly applied pressure of 1 MPa, the pedestal-shaped diaphragm exhibits a smaller deflection variation in its central region and a lower *PV* value. Although the sensitivity experiences a slight reduction, it remains above 6 μm/MPa, which meets the requirements of most application scenarios.

### SCCOF -FP sensing chip fabrication

**Fig. 3:**
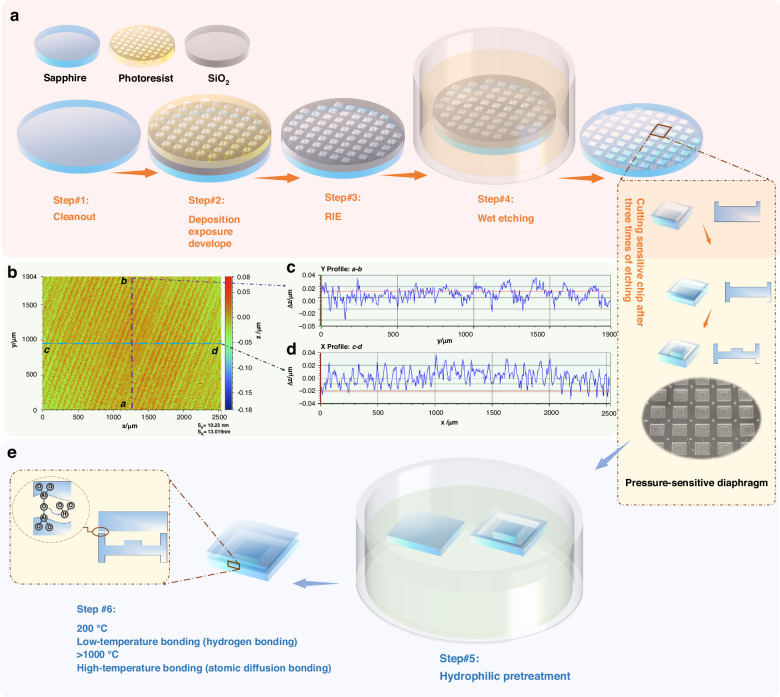
Sapphire sensing chip fabrication process. **a** Wet etching of sapphire, **b** measurement results of surface roughness of sapphire after etching using Profilometer, **c** roughness: Y Profile, **d** Roughness: X Profile, **e** direct bonding of sapphire

The surface reflectivity of the sensing chip directly influences its interference spectral characteristics and consequently the overall performance of the sensing system. The surface roughness of the etched sapphire is the most critical parameter determining its reflectivity. To ensure a high-quality surface finish, wet etching was employed, with the process flow illustrated in Fig. 3a. First, the sapphire substrate to be etched was cleaned using low-concentration HCl and NaOH solutions. A SiO₂ layer was then deposited on the sapphire substrate surface via Plasma-Enhanced Chemical Vapor Deposition (PECVD). After photoresist coating and exposure, the resist was removed, and the pattern was transferred to the SiO₂ hard mask using Reactive Ion Etching (RIE) technology. Subsequently, wet etching was performed to form the recessed features on the sapphire substrate. To balance the etch rate and surface roughness, an optimized recipe was established through systematic investigation: an etch temperature of 290 °C and a phosphoric-to-sulfuric acid volume ratio of 1:3. This resulted in a final etch rate of approximately 0.9 μm/min. Three sequential etching steps were performed on an initial 230 μm thick sapphire substrate. The first etching step, applied to the bottom surface, defined the pressure-sensitive surface with a depth of 25 μm. The second etching step, applied to the top surface, created the Fabry-Perot reference surface with a depth of 80 μm. The third etching step, also on the top surface, preserved a central 1.5 mm × 1.5 mm pedestal platform by etching the surrounding area to a depth of 25 μm, thereby forming the final pedestal structure. Figure 3b presents the measured surface roughness of the sapphire substrate after the etching process use Profilometer. The root mean square (RMS) height was determined to be 13.019 nm.

Following the etching process, the sapphire substrate containing multiple pressure-sensitive diaphragm arrays and the individual diaphragm wafers were bonded using a high-temperature wafer-level bonding technique. Subsequent dicing of this bonded stack yielded individual sensing chips. The high melting point and exceptional hardness of sapphire, coupled with the strong chemical bonds in α-Al_2_O_3_, necessitate the application of significant pressure to achieve intimate contact between the two surfaces during direct bonding. Furthermore, a high bonding temperature is required to form robust covalent bonds at the interface. Figure 3e shows a schematic of the bonding process for a single sensor chip. The etched pressure-sensitive diaphragm was first treated to deposit a hydrophilic hydroxyl layer (-OH) on its surface, facilitating the initial adhesion between the sapphire diaphragm and the sapphire substrate. The adhered sapphire chip was then placed in a bonder for pre-bonding at 200 °C. Finally, the chip was transferred to a high-temperature bonding furnace to complete the wafer-level bonding at the interface (>1000 °C).

### APSC-FFT algorithm

**Fig. 4:**
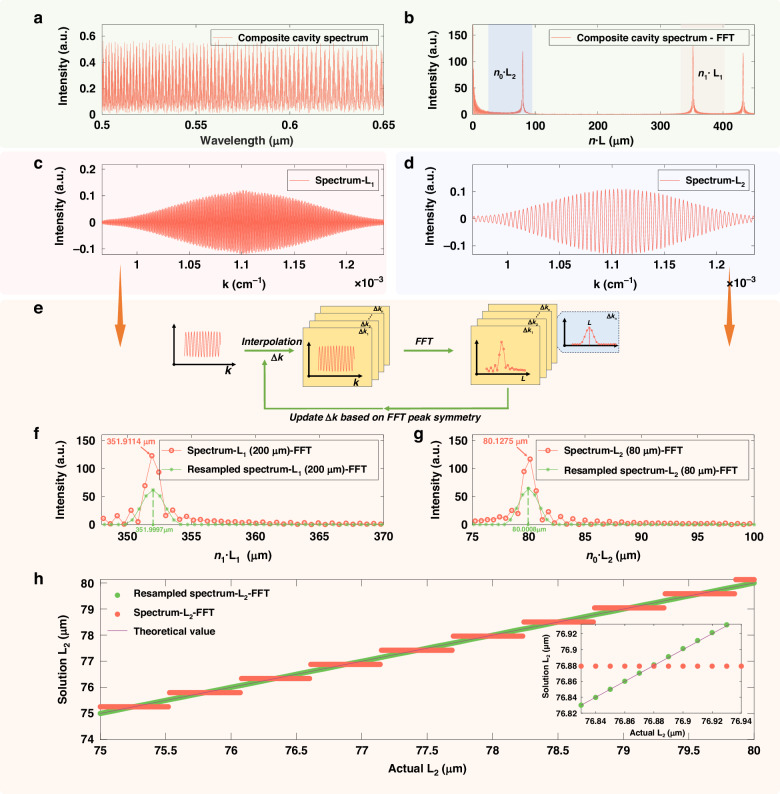
Simulation results of modified FFT algorithm with adaptive peak shape correction. **a** Original spectrum, **b** FFT result of original spectrum, **c** reinterpolated and windowed FP1 spectrum after filtering, **d** reinterpolated and windowed FP2 spectrum after filtering, **e** process of Modified FFT Algorithm, **f** FP1 spectral FFT comparison: before vs. after peak shape correction, **g** FP2 spectral FFT comparison: Before vs. after peak shape correction, **h** calculated L2 value comparison: before vs. after peak shape correction

The acquired spectral signal is discrete and sampled at equal wavelength intervals. Figure 4a shows the reflection spectrum of the sensing chip in its initial state, with simulation parameters set as *λ* = 0.5–0.65 μm, *n*_1_ = 1.76, *L*_1_ = 200 μm and *L*_2_ = 80 μm. To perform the Fourier transform, the spectrum must be re-sampled at equal wavenumber intervals prior to the transformation. Therefore, an interpolation step is applied to the acquired spectrum to convert it to equal wavenumber spacing before executing the Fourier transform. For algorithmic simplicity, linear interpolation was employed. Given that the spectrometer has *N* pixels, the pixel coordinates after interpolation are denoted as *n*_pk_ = 0, 1, 2 …, *N*-1:

4 $${k}_{\mathrm{int}}({n}_{pk})=\frac{2\pi }{{\lambda }_{N-1}}+\Delta k\cdot ({n}_{pk}-1)$$

In the equation, *k*_int_ represents the wavenumber coordinates to be interpolated at equal intervals, *λ*_*N*-1_ is the maximum wavelength value, and Δ*k* is the wavenumber interval. The discrete acquired spectrum is linearly interpolated using *k*_int_ as the new coordinate system, yielding an equally wavenumber-spaced sampled spectrum *I*_kout_(*k*_int_). The Fast Fourier Transform (FFT) result of the interpolated spectrum is shown in Fig. 4b. Since the horizontal axis of the FFT can be directly converted into optical path difference (OPD), the values of *L*_1_ and *L*_2_ can be determined from the peak positions in the FFT result.

Ideally, the Fourier transform of an infinite-length single-frequency cosine signal is a Dirac delta function, exhibiting a symmetric spectral peak. However, because the actual spectral signal consists of finite-length discrete data, the FFT suffers from limited resolution and spectral leakage. This leads to broadening of the Fourier spectral peak, peak shape distortion, and significant peak position estimation errors, as indicated by the red curves in Fig. 4f, g. To enhance the accuracy of FFT-based peak position estimation, we propose an adaptive peak-shift correction FFT algorithm. This algorithm adaptively adjusts the wavenumber interval Δ*k* in Eq. (4) based on an assessment of the symmetry of the FFT peak. It then re-interpolates the original spectrum at the new equal wavenumber intervals. This process ensures that the FFT peak becomes symmetric under the current sampling interval, which guarantees that the true gap length value coincides with a discrete sampling point of the FFT, thereby ensuring accurate peak localization.

The specific signal processing flow is as follows: First, the original spectrum is interpolated to equal wavenumber intervals and then subjected to FFT, with the result shown in Fig. 4b. Bandpass filters are then designed based on the peak positions corresponding to *L*₁ and *L*₂ in the FFT spectrum. Applying these filters isolates the individual cavity spectra for FP1 and FP2. A Hamming window is applied to each single-cavity spectrum to mitigate spectral leakage during the subsequent FFT, as shown in Fig. 4(c), d. Finally, FFT is performed on this processed spectrum, and its peak symmetry is evaluated. To simplify the algorithmic implementation for assessing peak symmetry, we employ a method based on calculating the intensity offset around the peak. The intensity values immediately preceding and following the peak are extracted to compute this offset, the magnitude of which directly indicates the degree of peak asymmetry. During the iterative calculation process, this offset is progressively minimized, leading to a gradual improvement in peak symmetry. (Supporting Information S6). The value of Δ*k* is iteratively adjusted, followed by re-interpolation and FFT. Once peak symmetry is achieved, the corresponding peak position directly yields the target gap length. As shown in Fig. 4(f), g (green curves), the peak position estimation errors for *L*₁ and *L*₂ are approximately 0.3 nm and 0.8 nm, respectively. A comparison between the error results after applying the iterative algorithm for multiple gap sets (*L*_1_ = 200 μm, *L*_2_ = 75-80 μm, Δ*L* = 10 nm) and the errors from direct FFT peak-searching is presented in Fig. 4h. The detailed iterative algorithm resulted in nearly a hundredfold reduction in the average error. The iterative simulation results for different gap values are shown in Fig. S2 (Supporting Information S2). The proposed method maintains the computational speed of the traditional FFT while significantly enhancing measurement precision, making it suitable for high-speed, high-precision measurement scenarios with stringent real-time requirements.

## Discussion

### Experiment system

**Fig. 5:**
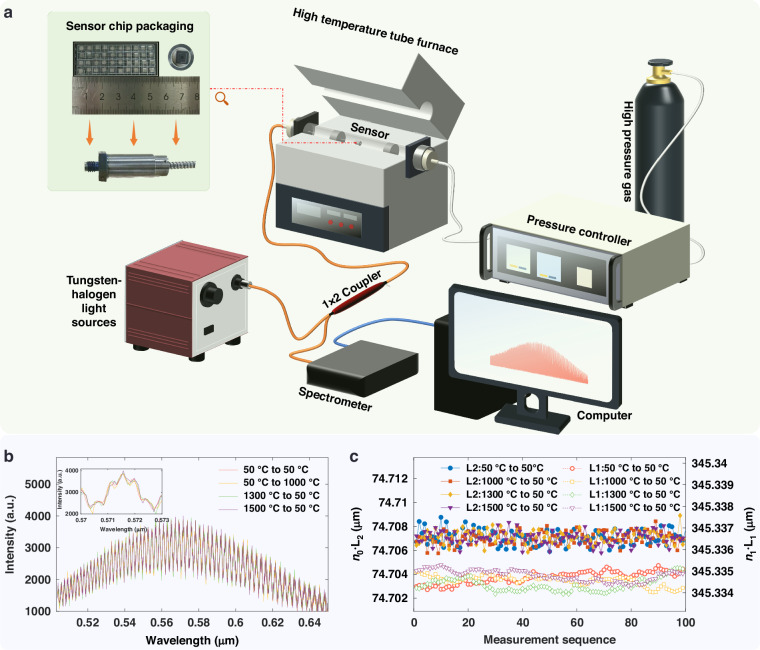
Schematic diagram of the experimental system and test signals. **a** sensor packaging and experimental system, **b** original spectrum of the sensing chip at 50 °C, **c** extracted cavity lengths of the sensing chip at 50 °C

The experimental setup is illustrated in Fig. 5a. Its main components include: a halogen tungsten lamp, a fiber optic coupler, the all-sapphire high-temperature pressure sensor, a tube furnace, a pressure control system, a spectrometer, and a host computer. The actual operating temperature of the high-temperature sapphire sensor is ultimately limited by the packaging and the temperature tolerance of the optical fiber. The sensor consists of a gold-coated fiber, a hermetically sealed metal housing, and a collimating lens. The entire assembly utilizes laser welding for high-temperature sealing. The gold-coated fiber is secured within a collimating sleeve, and the fiber end is stabilized by filling a V-groove with nano-silver paste. The housing is fabricated from titanium alloy TC4, and the collimating lens is made of quartz, ensuring high-temperature performance and feasibility. (The characteristics of the sensor packaging are detailed in Supplementary Information S5).

To validate the high-temperature endurance of the sensor chip itself, an unpackaged sapphire sensing chip was first annealed in a high-temperature furnace at 1000 °C, 1300 °C, and 1500 °C, respectively, for one day at each temperature. After cooling to a stable temperature of 50 °C, the chip was mounted in the packaging structure, and the system was used to measure the cavity lengths *L*_1_ (FP1) and *L*_2_ (FP2). The original signal and the extracted results are shown in Fig. 5b, c. The signal from the chip after high-temperature annealing remained robust, and the cavity lengths were virtually identical to those measured before annealing, demonstrating that the sensing chip can withstand temperatures up to 1500 °C. Packaging was performed after this high-temperature testing. The fully packaged sensor is shown in Fig. 5a. The packaged sensor was then installed in a pressure chamber, which was placed inside the tube furnace. The system utilizes a halogen lamp as the light source. The emitted light is directed through a 1 × 2 coupler to the sensor head, and the reflected light is collected by a custom-built spectrometer. The operational wavelength range of the spectrometer is 503.4568 –653.3530 nm, with a pixel resolution of 32.5 pm. Figure S3 (Supporting Information) presents a comparative analysis of the demodulation results for several sets of raw spectra acquired under different conditions, contrasting the outcomes before and after FFT peak-shape correction.

### Sensor calibrate

**Fig. 6:**
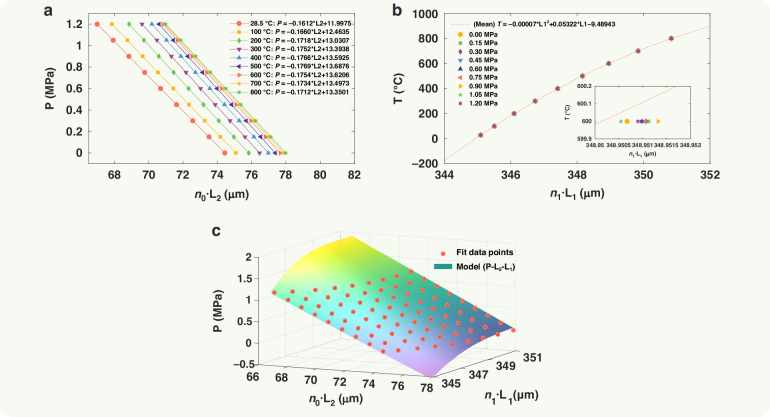
Sensor calibrate. **a** P- L2 calibration curve, **b** T- L1 calibration curve, **c** P- L1 -L2 calibration surface

Within the sensor’s pressure measurement range (0–1.2 MPa), nine calibration points were selected (0, 0.15, 0.3, 0.45, 0.6, 0.75, 0.9, 1.05, 1.2 MPa). Similarly, nine calibration points were chosen across the temperature range (28, 100, 200, 300, 400, 500, 600, 700, 800 °C). At each measurement point, the cavity lengths (*L*_1_, *L*_2_) were measured ten times, and the average values were used for sensor calibration. Figure 6a shows the variation of *L*_2_ with pressure at different temperatures. The pressure sensitivity of the sensing chip at room temperature was 6.203 μm/MPa. It is evident that both the pressure sensitivity and the initial cavity length of the sensing chip change with temperature.

Similarly, the extracted *L*_1_ at various measurement points are shown in Fig. 6b. It can be observed that the sapphire substrate thickness *L*_1_ increases solely with rising temperature. The data from the temperature calibration points were averaged and then fitted with a quadratic curve against *L*_1_ to obtain the *T*- *L*_1_ calibration equation. The systematic error for temperature measurement was 1.06 °C. With 800 °C as the full scale (F.S.), the overall accuracy of the temperature measurement was 0.13% F.S.

The inherent temperature information contained in the substrate cavity length *L*_1_ of the sensing chip enables in situ temperature compensation for pressure measurements. The data from the pressure calibration points, along with *L*_1_ and *L*_2_, were fitted with a high-order surface to derive the *P*-*L*_1_*-L*_2_ calibration equation, as shown in Fig. 6c. The systematic error for pressure measurement was 2.16 kPa. With 1.2 MPa as the full scale, the overall accuracy across the entire temperature range (0–800 °C) was 0.18% F.S.

### Sensor testing

**Fig. 7:**
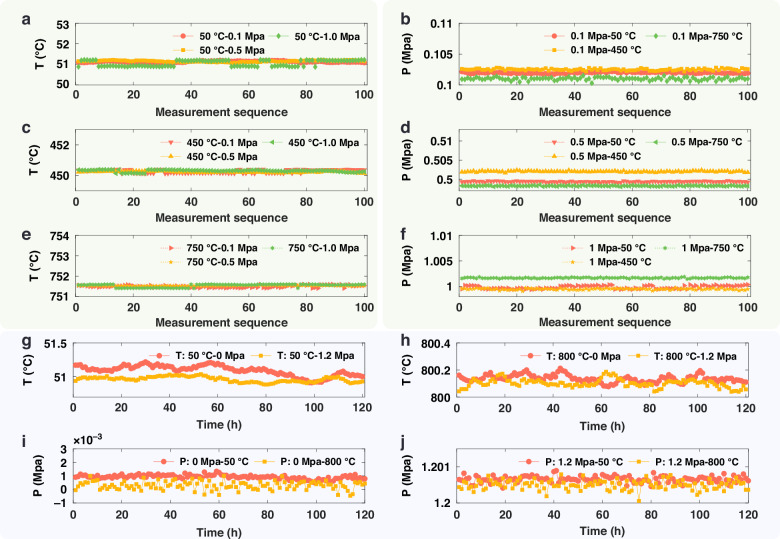
Sensor testing. **a** Temperature measurement repeatability test results at 50 °C, **b** pressure measurement repeatability test results at 0.1 MPa, **c** temperature measurement repeatability test results at 450 °C, **d** pressure measurement repeatability test results at 0.5 MPa, **e** temperature measurement repeatability test results at 750 °C, **f** pressure measurement repeatability test results at 1 MPa, **g** temperature measurement stability test results at 50 °C, **h** temperature measurement stability test results at 800 °C, **i** pressure measurement stability test results at 0 MPa, **j** pressure measurement stability test results at 1.2 MPa

To validate the performance of the sensor system, new test points, distinct from those used for calibration, were selected for multiple repeated measurements. The temperature test points were 50 °C, 450 °C, and 750 °C. The temperature measurement results are shown in Fig. 7a, c, e. The maximum error in the temperature data from 100 repeated measurements at each point was 1.61 °C, yielding an overall measurement accuracy of 0.2% F.S. The standard deviation, calculated using Bessel’s formula, was 0.07 °C, resulting in a temperature measurement repeatability of 0.02% (for a t-value at a 0.95 confidence level, t_0.95_).

The pressure test points were 0.1 MPa, 0.5 MPa, and 1 MPa. The results of a single pressure test series are shown in Fig. 7(b), d, f. The maximum error in the pressure data from 100 repeated measurements at each point was 2.79 kPa, yielding an overall measurement accuracy of 0.23% F.S. The standard deviation, calculated using Bessel’s formula, was 0.16 kPa, resulting in a pressure measurement repeatability of 0.03% (t_0.95_).

Furthermore, the stability of the pressure measurements from the proposed sapphire high-temperature pressure sensor was tested. Stability tests were conducted at both 50 °C and 800 °C for the sensor’s zero point (0 MPa) and full scale (1.2 MPa) over 120 h. The results, shown in Fig. 7g–j, demonstrate a temperature measurement stability better than 0.04% F.S. and a pressure measurement stability better than 0.12% F.S.

## Conclusion

This study successfully designed and validated an all-sapphire composite-cavity sensor system for simultaneous temperature and pressure measurement in extreme high-temperature environments. The fabricated all-sapphire sensor was systematically packaged and underwent rigorous calibration and performance testing across a broad temperature range (28 –800 °C) and pressure range (0–1.2 MPa). Experimental results confirmed that the sensing chip can withstand prolonged exposure to 1500 °C, with its performance remaining excellent after cooling down. The sensor system exhibits a temperature measurement systematic error better than 0.13% F.S., a pressure measurement systematic error better than 0.18% F.S., a temperature measurement stability better than 0.04% F.S., and a pressure measurement stability better than 0.12% F.S. The sensor system demonstrates outstanding measurement accuracy, stability, and reliability under the specified extreme temperature and pressure conditions. While the all-sapphire sensing chip itself can withstand temperatures up to 1500 °C, the overall high-temperature capability of the packaged sensor is currently limited by factors such as the optical fiber material and packaging techniques. Subsequent research aimed at addressing these related challenges is expected to lead to a further leap in the overall sensor performance.

## Materials and methods

In this study, the sensor fabrication employed a wet etching process. The procedure was as follows: the sapphire substrate was first cleaned using dilute HCl and NaOH solutions, followed by the deposition of a SiO₂ layer via plasma-enhanced chemical vapor deposition (PECVD). A hard mask was then fabricated by combining photolithography and reactive ion etching (RIE). Wet etching was subsequently carried out under optimized conditions (290 °C with a H₃PO₄ to H₂SO₄ volume ratio of 1:3) yielding an etch rate of approximately 0.9 μm/min. A three-step etching sequence was performed: etching the bottom surface by 25 μm to form the pressure-sensitive region, the top surface by 80 μm to define the Fabry–Perot reference surface, and the peripheral area of the top surface by 25 μm to leave a central pedestal measuring 1.5 mm × 1.5 mm. The resulting root mean square (RMS) surface roughness was measured to be 13.019 nm. After etching, the sapphire pressure-sensitive diaphragm and the substrate were integrated via high-temperature wafer-level bonding. Prior to bonding, a hydrophilic hydroxyl group (–OH) layer was formed on the etched surface to enhance adhesion. Pre-bonding was conducted at 200 °C, after which the assembly was transferred to a high-temperature bonding furnace for final bonding above 1000 °C, facilitating the formation of covalent bonds at the interface.

## Supplementary information

Supplementary Information_sub_R2
