# Supplementary material for: All-sapphire-based high-temperature pressure sensor system with in situ temperature compensation: innovative cavity design, fabrication, and APSC-FFT algorithm
Source: Microsyst Nanoeng. 2026 Apr 29;12:159. doi: 10.1038/s41378-026-01290-5 (PMC13125206; doi:10.1038/s41378-026-01290-5)
Supplement: Supplementary file 1 — Supplementary Information_sub_R2 [file 41378_2026_1290_MOESM1_ESM.docx]

**Supplementary Information**

All-Sapphire-Based High-Temperature Pressure Sensor System with In-Situ Temperature Compensation: Innovative Cavity Design, Fabrication, and APSC-FFT Algorithm

Jiahang Tan^1^, Feng Qin^1^, Ning Wang^1,^* , Zhiqiang Shao^2,^*, Jie Zhang^1^, Yong Zhu^1^

^1^College of Optoelectronic Engineering, Chongqing University, Chongqing 400044, China

^2^The 49th Research Institute of China Electronics Technology Group Corporation, Harbin 150000, China

* Ning Wang. *E-mail address:* [ningw@cqu.edu.cn](mailto:ningw@cqu.edu.cn)

* Zhiqiang Shao. *E-mail address:* szq880227@163.com

**S1: Reflection spectra of a single cavity with different PV**

The extent of diaphragm deformation is quantified by the Peak-to-Valley (PV, *PV* = *L*_max_ - *L*_min_) value of *L* within the effective spot area, as illustrated in Figs. S1. Reflection spectra of a single cavity were simulated for an initial air gap of 80 μm, with the *PV* variation modeled using a quadratic curve. The simulations demonstrate that the quality of the reflection spectrum deteriorates rapidly as the *PV* increases.


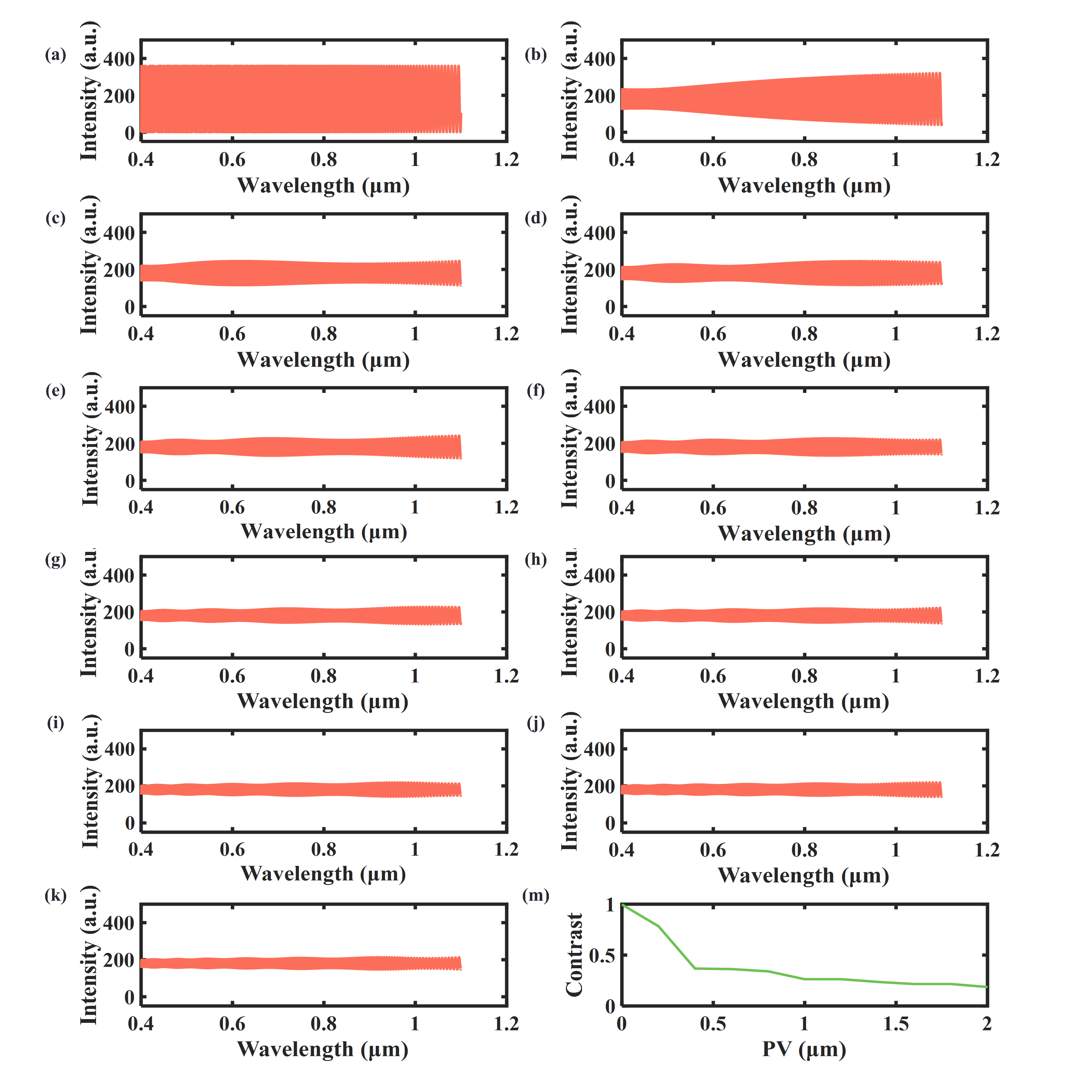

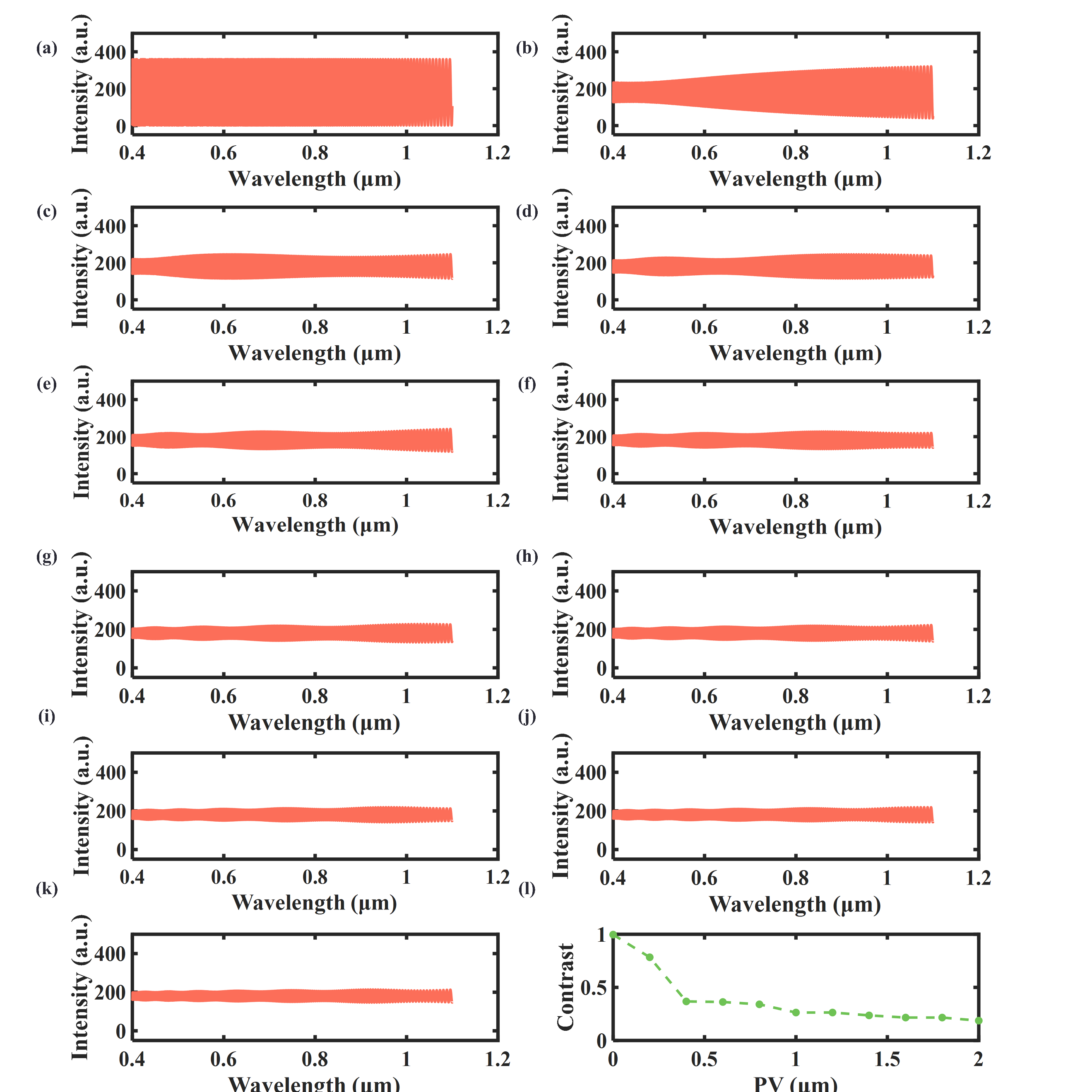


Fig. S1. (a) Deformation at PV = 0μm, (b) Deformation at PV = 0.2μm, (c) Deformation at PV = 0.4μm, (d) Deformation at PV = 0.6μm, (e) Deformation at PV = 0.8μm, (f) Deformation at PV = 1.0μm, (g) Deformation at PV = 1.2μm, (h) Deformation at PV = 1.4μm, (i) Deformation at PV = 1.6μm, (j) Deformation at PV = 1.8μm, (k) Deformation at PV = 2.0μm, (l) Contrast of light intensity.

**S2: Reflection spectra of a single cavity with different PV**

To demonstrate the proposed algorithm, a simulation was performed using the variable air-gap cavity as a specific example (*L*_2_ = 75-80 μm, Δ*L* = 0.01 nm). Selected corrected spectra from this simulation are presented in Fig. S2.


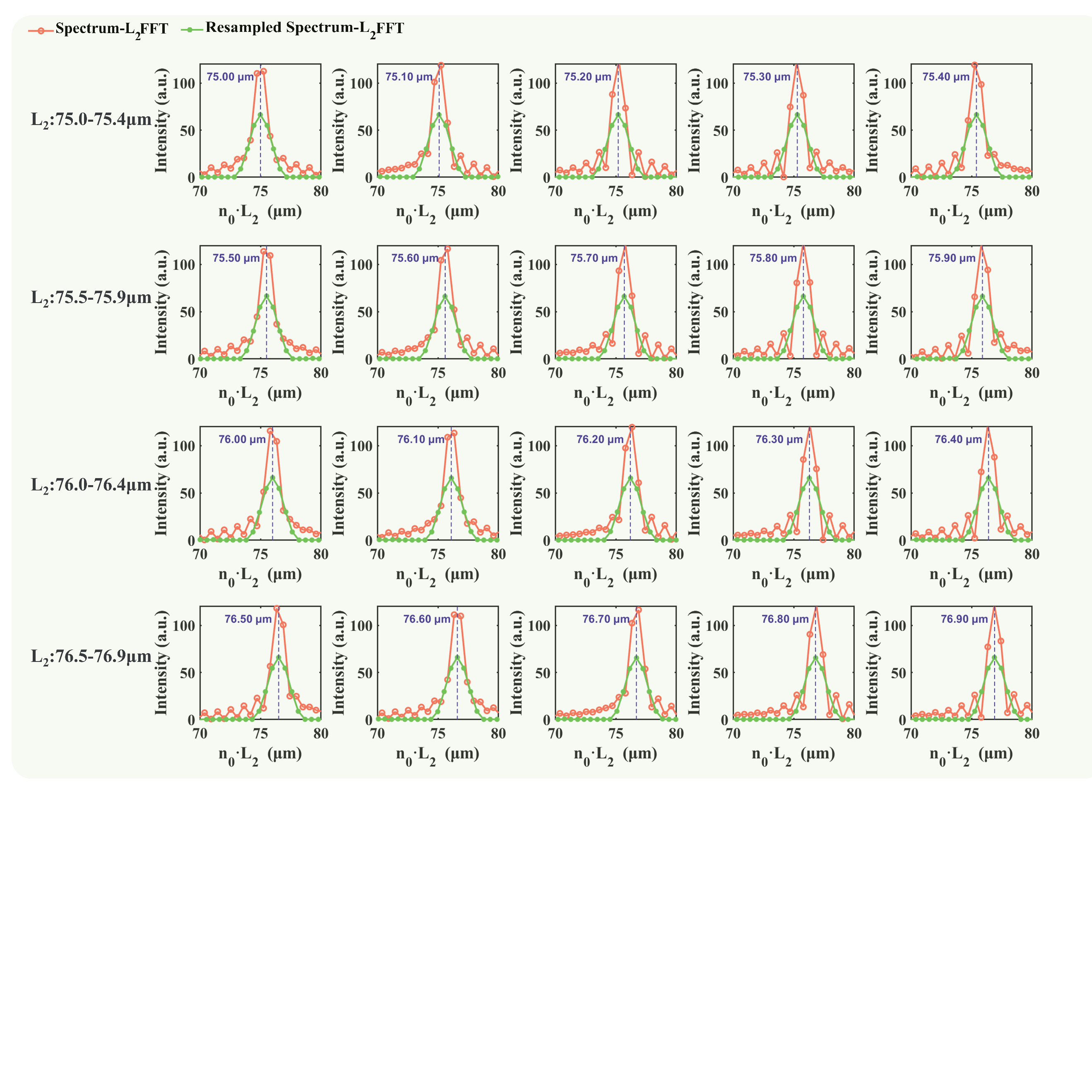

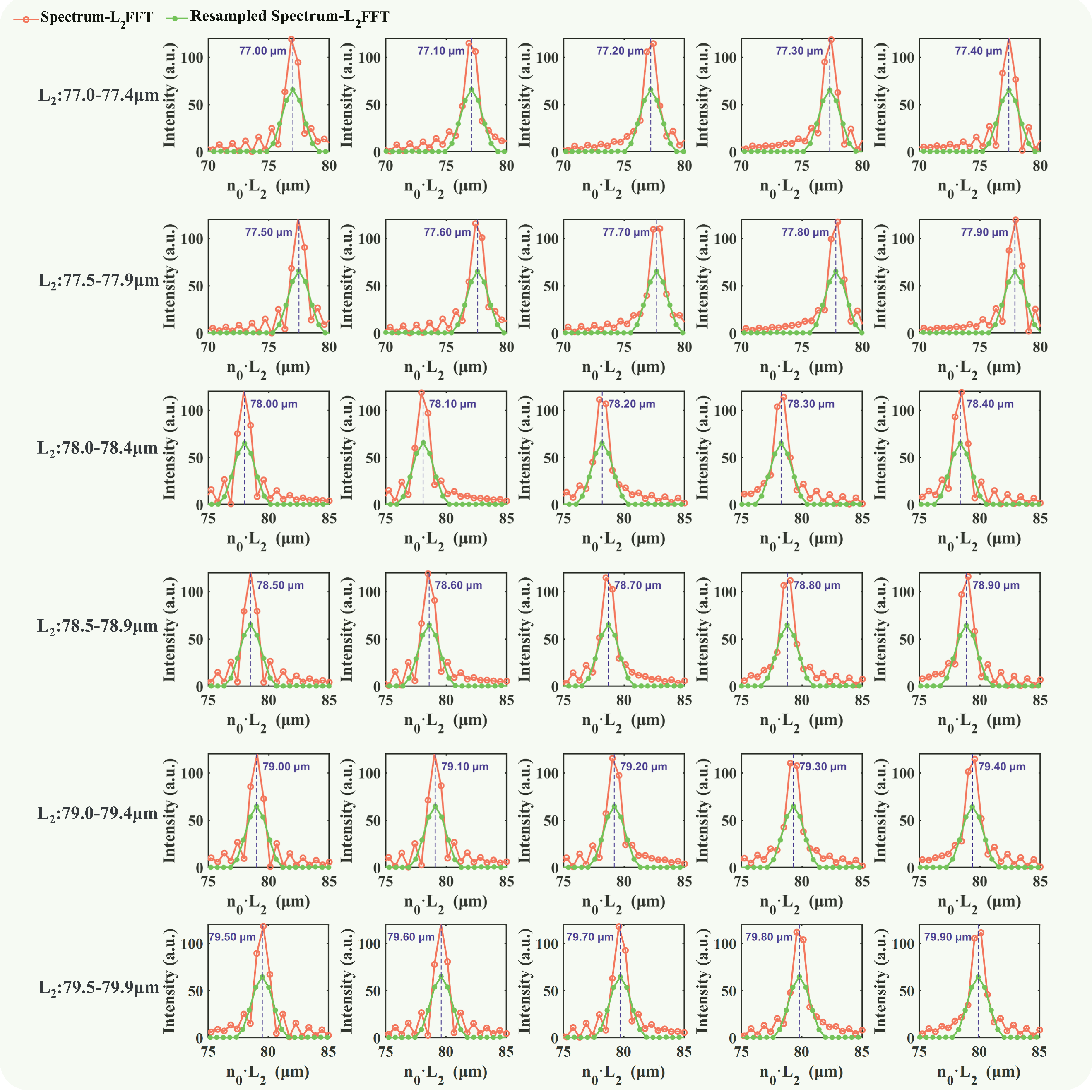


Fig. S2. Comparison of FFT results before and after peak correction

**S3:** **Comparison of raw spectra and FFT results at 10 temperature points**

Figure S3 displays ten sets of raw spectra measured from room temperature up to 800 °C, along with their corresponding demodulation results after applying the FFT peak-shape correction. The figure effectively demonstrates the significant improvement achieved by the correction algorithm.


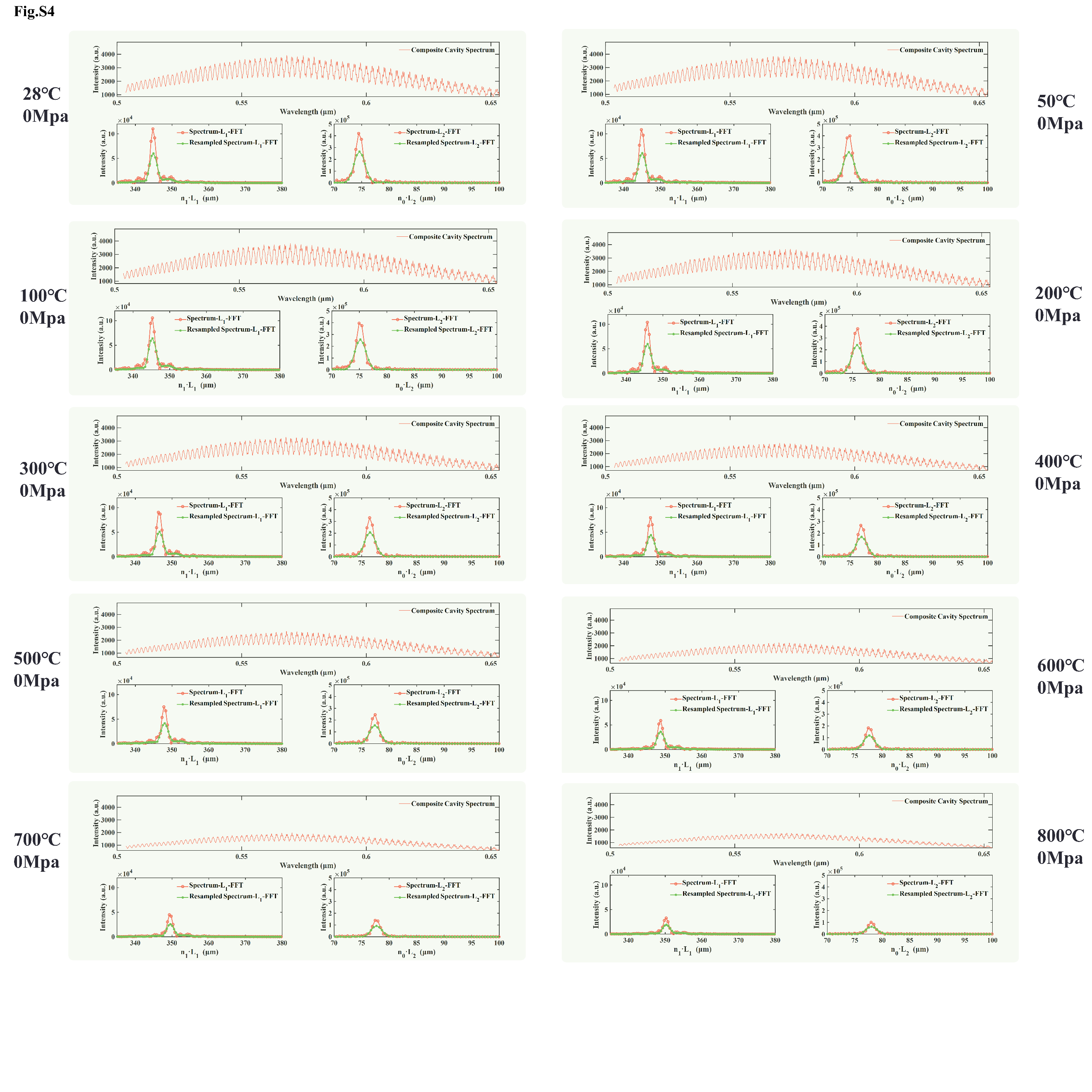

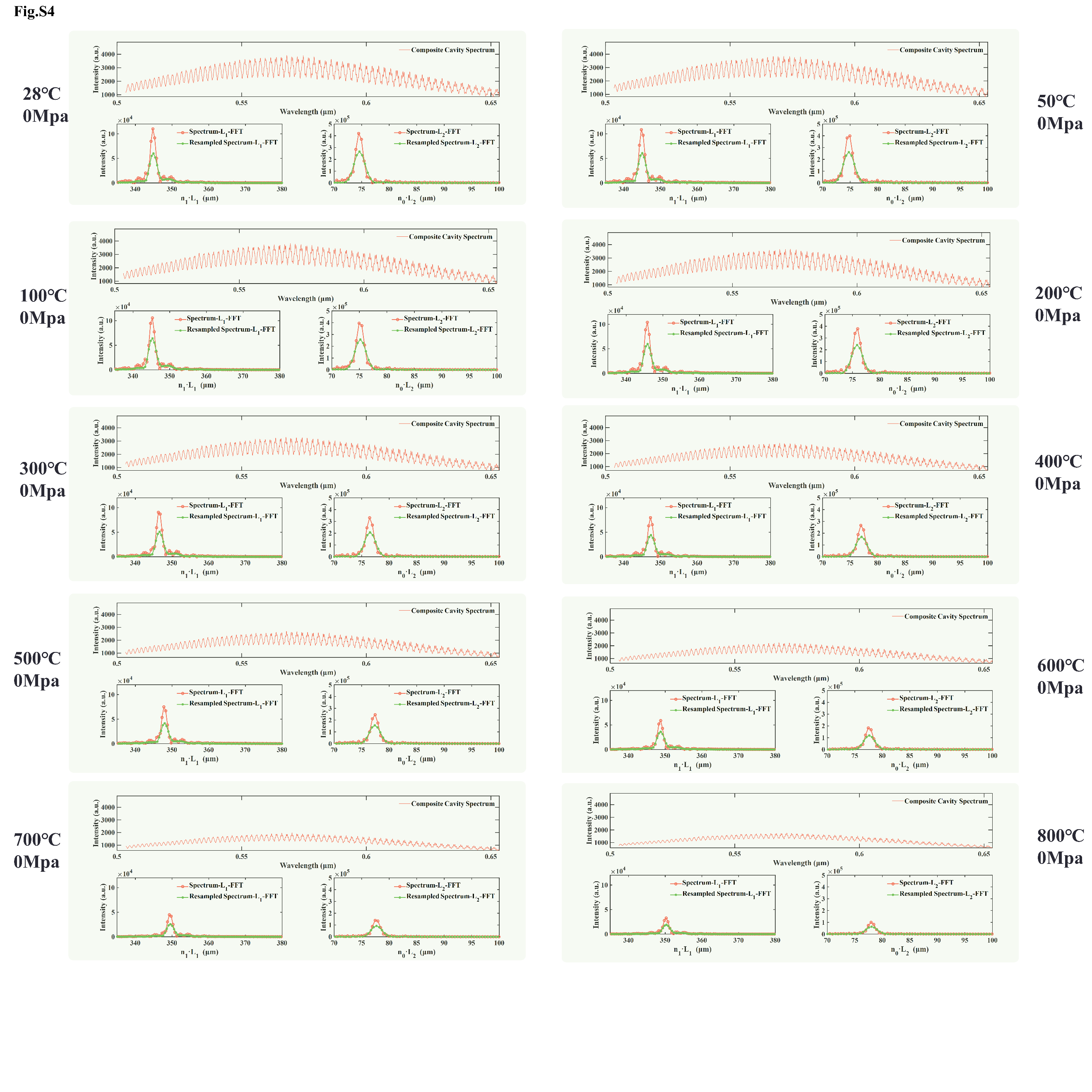


Fig. S3. Comparison of raw spectra and FFT results at 10 temperature points.

**S4:** **Analysis of Temperature-Pressure Compensation in the Sapphire Sensor**

As shown in Fig. S4(a), the composite-cavity sensor designed in this work consists of two key elements. FP1 is formed by the entire sapphire substrate, which has a uniform thickness exceeding 200 μm. This substrate exhibits minimal deformation under pressure, and the deformation of its top and bottom surfaces is essentially identical. Therefore, the FP1 cavity length, defined by the substrate thickness, is largely insensitive to pressure. However, its length changes with temperature due to the thermal expansion of sapphire. FP2 is primarily formed by the air gap between the bottom surface of the sapphire substrate and the sapphire pressure-sensitive diaphragm. Changes in ambient pressure alter the FP2 cavity length through the diaphragm's deflection and the substrate's minor deformation. Similarly, temperature changes affect the FP2 cavity length via the thermal expansion of the diaphragm, substrate, and the air within the gap.


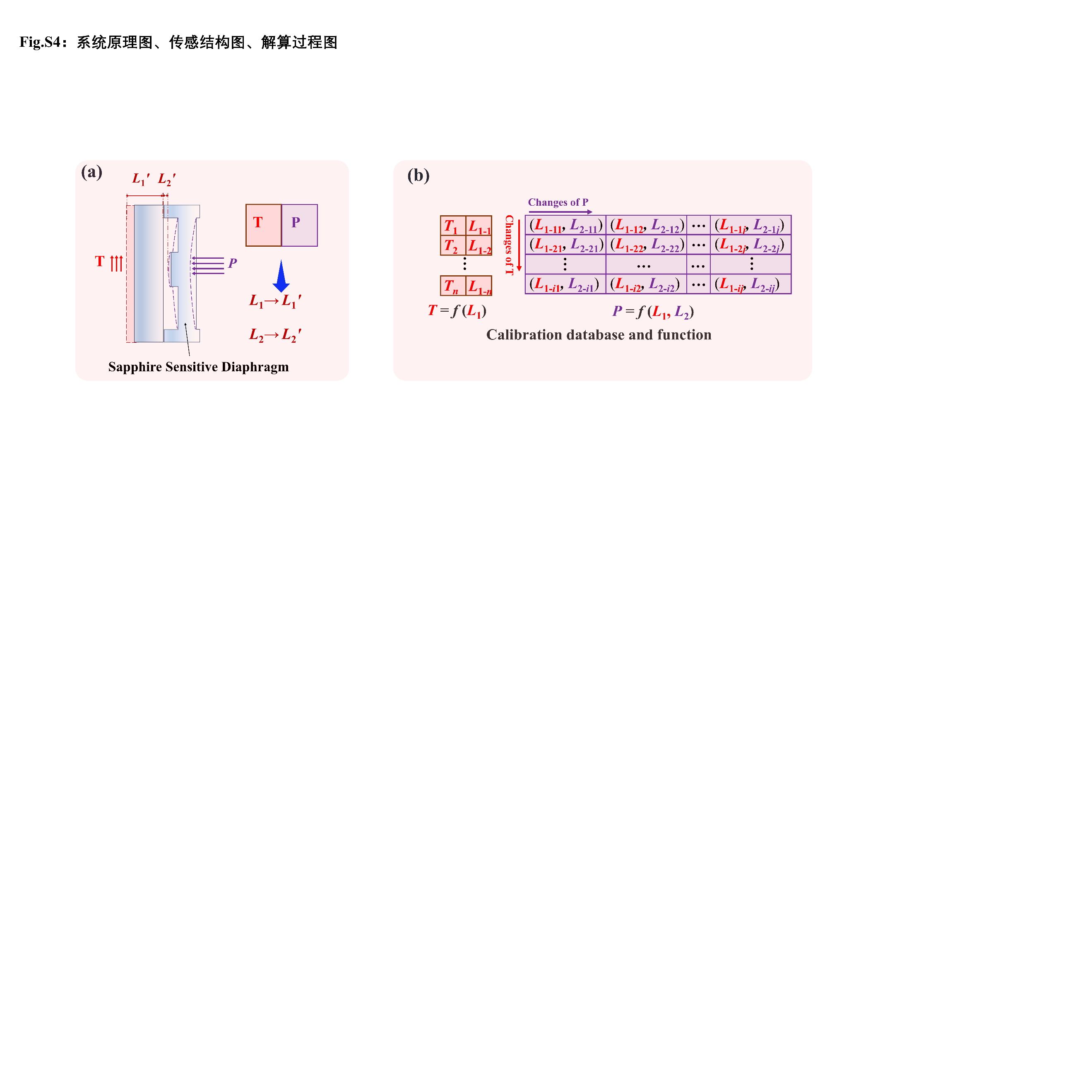


Fig. S4. Schematic of the sensor's temperature compensation process. (a) Schematic of sensor temperature-pressure sensitivity, (b) Schematic of the sensor's temperature-pressure demodulation process.

**S5: High-Temperature Packaging Analysis of the Sapphire Pressure Sensor**

The fundamental configuration of the sensor designed in this work is shown in Fig. S5. It consists of a gold-coated fiber, a hermetically sealed metal housing, and a collimating lens. The overall sensor employs laser welding for high-temperature sealing. The gold-coated fiber is secured within a collimating sleeve. The housing is made from titanium alloy TC4, and the collimating lens is quartz, ensuring high-temperature performance and feasibility. During long-term high-temperature testing, no significant degradation was observed in any component, and the sensor signal remained robust. Furthermore, a high-temperature-tolerant gold-coated fiber was selected for signal transmission. A double-sleeve collimator structure is used to secure the fiber, minimizing microbending or stress induced by external forces. The sensing fiber is externally protected by an armored cable with an outer layer to reduce environmental interference. As shown in Fig. S5(a), the probe end of the gold-coated fiber is fixed using a crimping process. V-grooves at both ends of the sleeve are filled with nano-silver paste, and the probe end is polished to complete the fixation. The overall sensor installation schematic is shown in Fig. S5(c). The sensing chip contacts the measured medium, operating in a high-temperature, high-pressure environment. The remaining parts, including the optical transmission path and packaging, are located in a temperature-attenuated zone where the ambient temperature is significantly lower, further ensuring the stability of the overall sensor package.


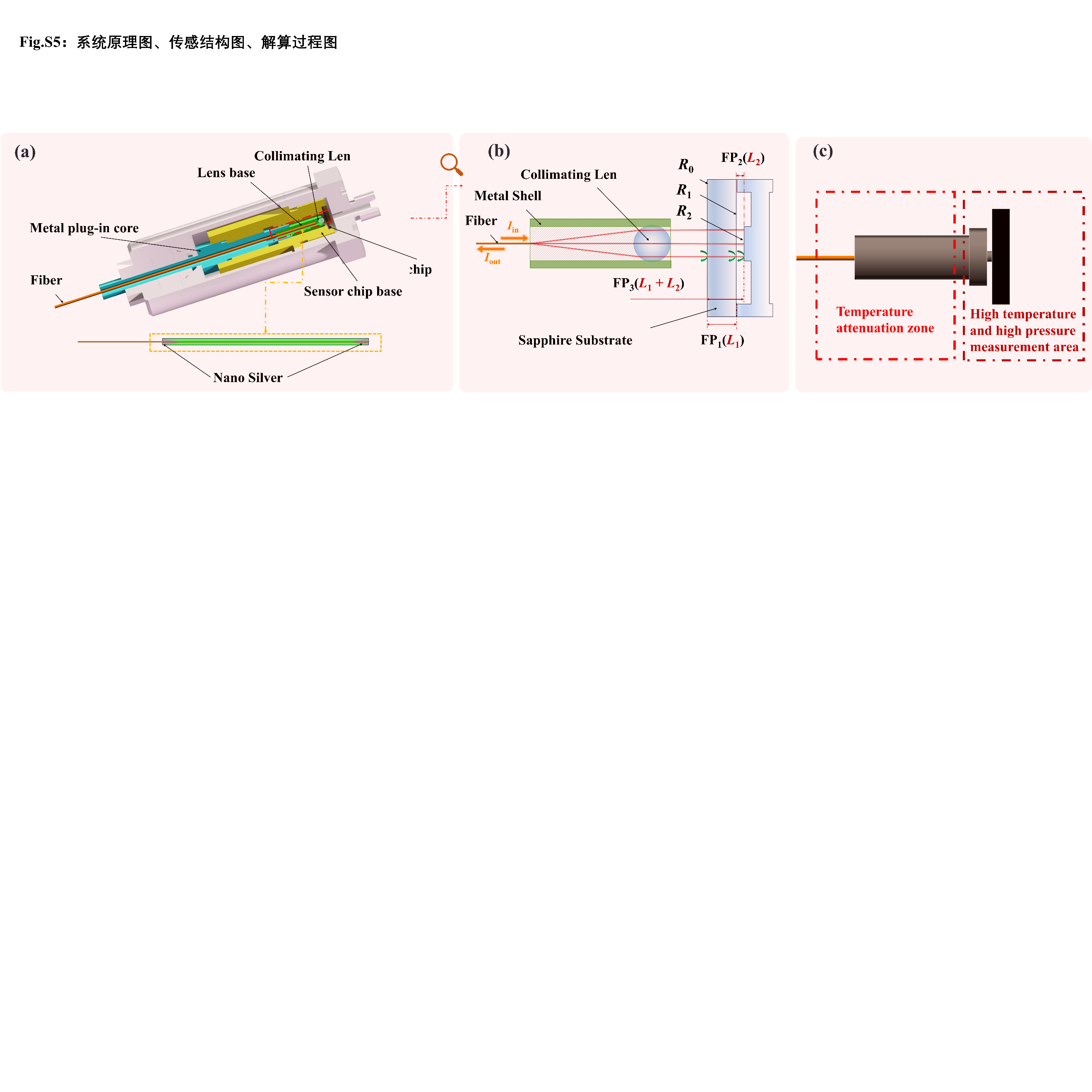


Fig.S5. (a) Schematic of the sensor packaging structure, (b) Schematic of the sensor optical path, (c) Schematic of the sensor installation.

**S6:** **Judgement of FFT Peak Symmetry**

The algorithms for extracting the frequency peak and the inverse-transform phase from the FFT result primarily suffer from errors under ideal conditions due to the discrete nature of the FFT frequency points for a discrete signal. This discretization leads to a discrete representation of the optical path difference, resulting in low resolution and significant errors. When the spectral frequency falls between the discrete FFT frequency points, the FFT spectral peak exhibits a shift. If the resampled spectral frequency can be made to precisely coincide with an FFT frequency point, the accuracy of frequency estimation and inverse-transform phase retrieval can be greatly enhanced. To simplify the algorithmic implementation for assessing peak symmetry, we employ a method based on the intensity offset around the peak. The intensity values before and after the peak are extracted, and the peak offset is calculated using Formula S1. The magnitude of this offset directly indicates the degree of peak symmetry. During the iterative calculation process, this offset progressively decreases, and the peak symmetry is gradually improved

$bais=\frac{x_{p+1}-x_{p-1}}{\max[(x_{p}-x_{p+1}),(x_{p}-x_{p-1})]}$ (S1)


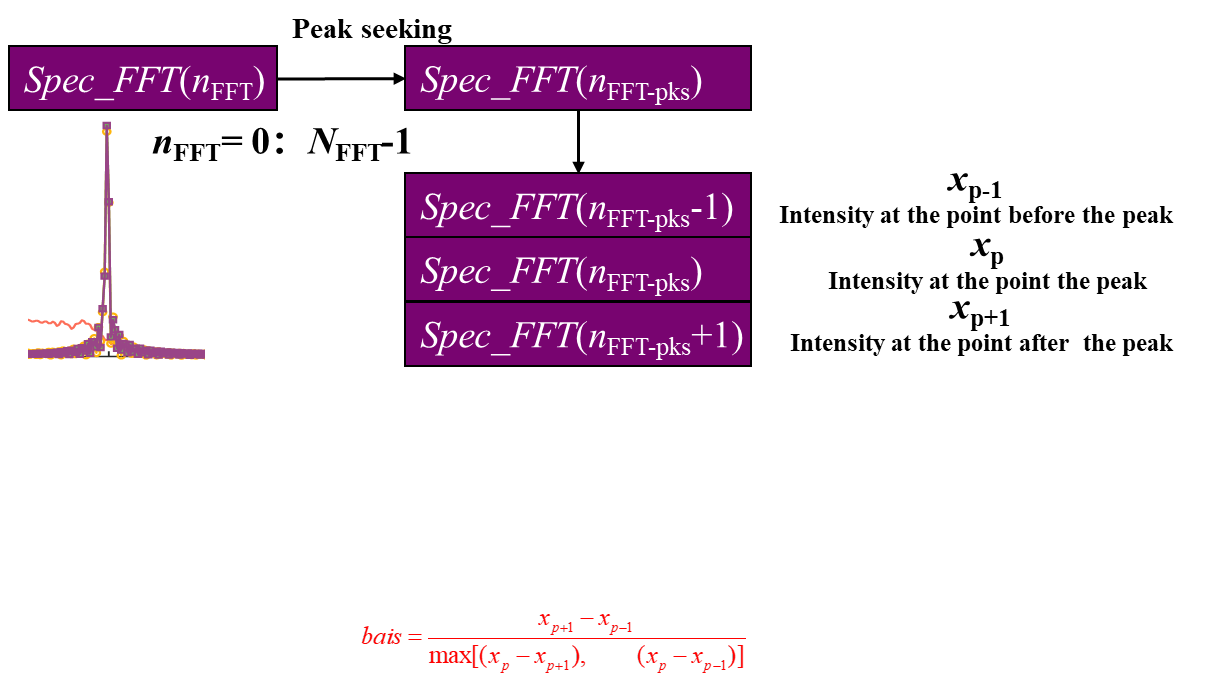


Fig.S6. Schematic of the Peak Symmetry Judgment Algorithm

**S7:** **The optical transmission model**

The reflection spectrum is the superposition of the amplitudes of the reflected light from each reflective surface. The reflected optical intensity is given by:

$I_{\text{out}}(\lambda)=\left|E_{01}(\lambda)+E_{12}(\lambda)+E_{23}(\lambda)\right|^{2}$ (S2)

In the equation, *λ* is the wavelength of the light source, *I*_out_ is the composite cavity’s reflection spectrum. *E*_0_ denotes the amplitude of the incident light.

$E_{01}(\lambda)=\sqrt{R_{0}(\lambda)}E_{0}(\lambda)e^{-i\pi}$ (S3)

 (S4)

 (S5)

When the incident light intensity is *I*_in_, the reflected optical intensity is given by:

$\begin{matrix} I_{\text{out}}(\lambda)=\left|E_{01}(\lambda)+E_{12}(\lambda)+E_{23}(\lambda)\right|^{2} \\ =(\sqrt{R_{0}(\lambda)}E_{0}(\lambda)e^{-i\pi}+ \\ (1-R_{0}(\lambda))\sqrt{R_{0}(\lambda)}E_{0}(\lambda)e^{-\text{i}\frac{4\pi n_{1}(\lambda)L_{1}}{\lambda}}+ \\ (1-R_{0}(\lambda))^{2}\sqrt{R_{0}}E_{0}(\lambda)e^{-\text{i(}\frac{4\pi n_{1}(\lambda)L_{1}}{\lambda}+\frac{4\pi n_{0}L_{2}}{\lambda}+\pi)})\bullet\\ (\sqrt{R_{0}(\lambda)}E_{0}(\lambda)e^{i\pi}+ \\ (1-R_{0}(\lambda))\sqrt{R_{0}(\lambda)}E_{0}(\lambda)e^{-\text{i}\frac{4\pi n_{1}(\lambda)L_{1}}{\lambda}}+ \\ (1-R_{0}(\lambda))^{2}\sqrt{R_{0}(\lambda)}E_{0}(\lambda)e^{\text{i(}\frac{4\pi n_{1}(\lambda)L_{1}}{\lambda}+\frac{4\pi n_{0}L_{2}}{\lambda}+\pi)}) \\ \\ =R_{0}(\lambda)(E_{0}(\lambda))^{2}+ \\ R_{0}(\lambda)(1-R_{0}(\lambda))(E_{0}(\lambda))^{2}e^{\text{i(}\frac{4\pi n_{1}(\lambda)L_{1}}{\lambda}-\pi)}+ \\ \sqrt{R_{0}(\lambda)}(1-R_{0}(\lambda))^{2}\sqrt{R_{0}(\lambda)}(E_{0}(\lambda))^{2}e^{\text{i(}\frac{4\pi n_{1}(\lambda)L_{1}}{\lambda}+\frac{4\pi n_{0}L_{2}}{\lambda})}+ \\ (1-R_{0}(\lambda))^{2}R_{0}(\lambda)(E_{0}(\lambda))^{2}+ \\ R_{0}(\lambda)(1-R_{0}(\lambda))(E_{0}(\lambda))^{2}e^{\text{i(}\pi-\frac{4\pi n_{1}(\lambda)L_{1}}{\lambda})}+ \\ (1-R_{0}(\lambda))^{3}\sqrt{R_{0}(\lambda)}\sqrt{R_{0}(\lambda)}(E_{0}(\lambda))^{2}e^{\text{i(}\frac{4\pi n_{0}L_{2}}{\lambda}+\pi)}+ \\ (1-R_{0}(\lambda))^{4}R_{0}(\lambda)(E_{0}(\lambda))^{2}+ \\ \sqrt{R_{0}(\lambda)}(1-R_{0}(\lambda))^{2}\sqrt{R_{0}(\lambda)}(E_{0}(\lambda))^{2}e^{\text{i(}-\frac{4\pi n_{1}(\lambda)L_{1}}{\lambda}-\frac{4\pi n_{0}L_{2}}{\lambda})}+ \\ (1-R_{0}(\lambda))^{3}\sqrt{R_{0}(\lambda)}\sqrt{R_{0}(\lambda)}(E_{0}(\lambda))^{2}e^{\text{i(}-\frac{4\pi n_{0}L_{2}}{\lambda}-\pi)} \\ \\ =R_{0}(\lambda)I_{\text{in}}+(1-R_{0}(\lambda))^{2}R_{0}(\lambda)I_{\text{in}}+(1-R_{0}(\lambda))^{4}R_{2}I_{\text{in}}+ \\ R_{0}(\lambda)(1-R_{0}(\lambda))I_{\text{in}}(2\cos(\frac{4\pi n_{1}(\lambda)L_{1}}{\lambda}-\pi))+ \\ (1-R_{0}(\lambda))^{3}\sqrt{R_{0}(\lambda)}\sqrt{R_{0}(\lambda)}I_{\text{in}}(2\cos(\frac{4\pi n_{0}L_{2}}{\lambda}+\pi))+ \\ \sqrt{R_{0}(\lambda)}(1-R_{0}(\lambda))^{2}\sqrt{R_{0}(\lambda)}I_{\text{in}}(2\cos(\frac{4\pi n_{1}(\lambda)L_{1}}{\lambda}+\frac{4\pi n_{0}L_{2}}{\lambda})) \\ =R_{0}I_{\text{in}}+(1-R_{0})^{2}R_{0}I_{\text{in}}+(1-R_{0})^{4}R_{0}I_{\text{in}}+ \\ R_{0}(1-R_{0})I_{\text{in}}(2\cos(\frac{4\pi n_{1}L_{1}}{\lambda}-\pi))+ \\ (1-R_{0})^{3}R_{0}I_{\text{in}}(2\cos(\frac{4\pi n_{0}L_{2}}{\lambda}+\pi))+ \\ (1-R_{0})^{2}R_{0}I_{\text{in}}(2\cos(\frac{4\pi n_{1}L_{1}}{\lambda}+\frac{4\pi n_{0}L_{2}}{\lambda})) \end{matrix}$ (S6)

**S8: Loading-Unloading Experimental Results for Sensor Calibration**

Loading and unloading experiments for both temperature and pressure were conducted at all test points, as shown in Fig. S7. Pressure loading-unloading experiments were performed at various temperatures, and temperature loading-unloading experiments were conducted under different pressures. The experimental results demonstrate that the sensor exhibits minimal hysteresis error and excellent recoverability.





Fig.S7 Sensor calibration experimental results. (a) Pressure loading-unloading cycles, (b) Temperature loading-unloading cycles.
